# Early change of plasma Epstein-Barr virus DNA load and the viral lytic genome level could positively predict clinical outcome in recurrent or metastatic nasopharyngeal carcinoma receiving anti-programmed cell death 1 monotherapy

**DOI:** 10.1186/s12885-024-12564-4

**Published:** 2024-07-03

**Authors:** Shaoyan Lin, Huaqiang Zhou, Gang Chen, Jinhui Xue, Qianwen Liu, Jianing Li, Yanhua Yang, Yuanyuan Zhao, Hua Bao, Yan Huang, Yuxiang Ma, Hongyun Zhao

**Affiliations:** 1 Department of Clinical Research, State Key Laboratory of Oncology in South China, Guangdong Key Laboratory of Nasopharyngeal Carcinoma Diagnosis and Therapy, Sun Yat-sen University Cancer Center, Guangdong Provincial Clinical Research Center for Cancer, Guangzhou, 510060 P. R. China; 2 Department of Medical Oncology, State Key Laboratory of Oncology in South China, Guangdong Key Laboratory of Nasopharyngeal Carcinoma Diagnosis and Therapy, Sun Yat-sen University Cancer Center, Guangdong Provincial Clinical Research Center for Cancer, Guangzhou, 510060 P. R. China; 3 Geneseeq Research Institute, Nanjing Geneseeq Technology Inc, Nanjing, China

**Keywords:** NPC, EBV, Immunotherapy, Biomarkers

## Abstract

**Purpose:**

Patients with recurrent or metastatic nasopharyngeal carcinoma (RM-NPC) have proven benefit from anti-programmed cell death 1 (anti-PD-1) monotherapy. Here, we retrospectively analyze the association of plasma Epstein-Barr virus (EBV) DNA load and tumor viral lytic genome with clinical outcome from 2 registered phase I trials.

**Methods:**

Patients with RM-NPC from Checkmate 077 (nivolumab phase I trial in China) and Camrelizumab phase I trial between March 2016 and January 2018 were enrolled. Baseline EBV DNA titers were tested in 68 patients and EBV assessment was performed in 60 patients who had at least 3 post-baseline timepoints of EBV data and at least 1 post-baseline timepoint of radiographic assessment. We defined “EBV response” as 3 consecutive timepoints of load below 50% of baseline, and “EBV progression” as 3 consecutive timepoints of load above 150% of baseline. Whole-exome sequencing was performed in 60 patients with available tumor samples.

**Results:**

We found that the baseline EBV DNA load was positively correlated with tumor size (spearman *p* < 0.001). Both partial response (PR) and stable disease (SD) patients had significantly lower EBV load than progression disease (PD) patients. EBV assessment was highly consistent with radiographic evaluation. Patients with EBV response had significantly improved overall survival (OS) than patients with EBV progression (log-rank *p* = 0.004, HR = 0.351 [95% CI: 0.171–0.720], median 22.5 vs. 11.9 months). The median time to initial EBV response and progression were 25 and 36 days prior to initial radiographic response and progression, respectively. Patients with high levels of EBV lytic genomes at baseline, including BKRF2, BKRF3 and BKRF4, had better progression-free survival (PFS) and OS.

**Conclusion:**

In summary, early clearance of plasma EBV DNA load and high levels of lytic EBV genes were associated with better clinical outcome in patients with RM-NPC receiving anti-PD-1 monotherapy.

**Supplementary Information:**

The online version contains supplementary material available at 10.1186/s12885-024-12564-4.

## Background

Nasopharyngeal carcinoma (NPC) is a head and neck cancer endemic to southern China and Southeast Asia [1]. Patients with recurrent or metastatic NPC (RM-NPC) have limited effective treatment options and poor clinical outcome [2]. With the development of immune-checkpoint inhibitors (ICIs), patients with RM-NPC have obtained significant clinical benefit [3–5]. In RM-NPC patients who had progressed on first-line treatment, anti-programmed cell death 1 (anti-PD-1) monotherapy showed promising antitumor activity, with objective response rates (ORRs) of 13–43% and median duration of response longer than 8 months [6]. However, thorough analysis of the connection of plasma and tissue Epstein–Barr virus (EBV) biomarkers with ICI therapy outcome is lacking [7, 8].

EBV infection is highly associated with NPC [9]. Latent infection is the predominant program of EBV infection in NPC, in contrast to lytic infection, which is the default mode of EBV infection in normal epithelium [10]. Type II latency of EBV infection is commonly observed in NPC, with the expression of various latent EBV genes, including LMP1, LMP2A, BARTs, EBNA1, and EBERs [11]. Expression of latency II EBV genes, notably LMP1, alters multiple cellular pathways and drives NPC pathogenesis [12, 13]. In comparison, the expression of lytic EBV genes is detected in small islets of NPC cells [14]. The BKRF2, BKRF3 and BKRF4 genes are lytic EBV genes located close to one another in the viral genome [15]. The BKRF2 gene is a true-late lytic gene during EBV reactivation which encodes glycoprotein L [16]. The BKRF3 and BKRF4, considered as early lytic genes, encode uracil-DNA glyosylase and a tegument protein, respectively. The BKRF3 and BKRF4 are direct transcriptional targets of Rta and Zta, two transactivators which trigger viral reactivation [17, 18]. However, little is known about the impact of BKRF2, BKRF3 and BKRF4 genes in NPC.

The role of plasma EBV DNA as a biomarker for patients with RM-NPC receiving anti-PD-1 therapy has been reported. However, controversial results were found in different studies. Notably, the association of early clearance of plasma EBV DNA with clinical outcome in RM-NPC patients treated with anti-PD-1 immunotherapy was positive in POLARIS-02 and CAPTAIN-1st trials but negative in the Mayo Clinic Phase 2 Consortium study of nivolumab [3, 5, 19, 20]. Moreover, the value of circulating viral DNA titer for distinguishing patients with RM-NPC receiving anti-PD-1 monotherapy who could obtain long-term clinical benefit has not been comprehensively investigated. Given the controversial evidence, more studies are needed to verify the use of EBV surveillance for RM-NPC patients treated with anti-PD-1 monotherapy.

In this study, we aimed to explore the role of plasma and tissue EBV biomarkers, including plasma EBV DNA load, tumor neoantigen burden (TNB), lytic EBV genes and EBV strains, for predicting RM-NPC patients’ response to anti-PD-1 monotherapy by analyzing the data from two phase I clinical trials evaluating camrelizumab and nivolumab.

## Methods

The current study was approved by the institutional ethics committee of Sun Yat-sen University Cancer Center and written informed consent were obtained from all patients. The study methods referred partially to our previous report, which detailly analyzed the survival outcomes and the association between copy number loss in either GZMB or GZMH genes and survival within the same population as this present study [21].

### Study design and patients

The design and results of Camrelizumab phase I trial and Checkmate 077 (nivolumab phase I trial in China) from March 2016 to January 2018 (ClinicalTrials.gov identifiers: NCT02721589 and NCT02593786) have been previously reported [4, 22]. Patients with RM-NPC from these 2 clinical trials were enrolled in current study. The distribution of treatments and the screen process of participants in current study were displayed in supplementary Figure S1 [see supplementary file]. The baseline tumor tissues and blood samples were obtained before anti-PD-1 immunotherapy.

Responses were evaluated by investigators using Response Evaluation Criteria in Solid Tumors (RECIST) version 1.1 at baseline and approximately every 6 weeks. Durable clinical benefit (DCB) was calculated as the percentage of patients who achieved complete response (CR) or partial response (PR) or stable disease (SD) lasted more than 6 months; non-durable clinical benefit (NDB) was defined as progression disease (PD) or SD lasted 6 months or less. Progression-free survival (PFS) was defined as the duration from the first treatment to PD or death. OS was calculated as the time from first dose to death.

### Sample collection and analysis

Plasma EBV DNA level was measured by real-time quantitative polymerase chain reaction (PCR) with probes against EBV genes before and every 2 weeks until disease progression. Real-time quantitative polymerase chain reaction was conducted using the ABI Prism 7500 Sequence Detection System from Applied Biosystems. The reagents for the DNA extraction and detection kit were commercially available assays provided by Sansure Biotech. This particular DNA extraction and detection kit has obtained approval from both the National Medical Products Administration and Conformité Européenne. For EBV assessment, we defined “EBV response” as 3 consecutive timepoints of load below 50% of baseline, and “EBV progression” as 3 consecutive timepoints of load above 150% of baseline.

Whole-exome Sequencing (WES) was performed in 60 patients with available tumor samples on Illumina HiSep4000 platform (Illumina, USA). QIAamp DNA FFPE Tissue Kit and DNeasy Blood and tissue kit (Qiagen, USA) were used to extract DNA from Formalin-fixed, paraffin-embedded (FFPE) or biopsy tumor tissues and blood samples, respectively. Genomic DNA was then quantified by a Qubit Fluorometer (Invitrogen) using the dsDNA HS Assay Kit (ThermoFisher Scientific, USA). The sequence reads were aligned to the human reference genome (hg19) using BWA-mem [23]. Somatic Single Nucleotide Variant and insertion/deletions were called with Mutect [24] and Scalpel [25], respectively. TNB referred to the number of tumor-mutated antigens. The association between EBV lytic genes read counts and clinical outcomes (DCB vs. NDB) were displayed by heatmaps. Levels of BKRF2, BKRF3 and BKRF4 read counts were grouped binarily as high versus low by median. As for assembly of EBV genome, all sequencing reads were aligned to hg19 using Browtie2 and human sequences were removed. Following the elimination of human sequences, the remaining reads underwent assembly via SOAPdenovo [26]. SOAPdenovo amalgamated overlapping reads utilizing the de Bruijn graph algorithm to produce contigs. The paired-end information was then leveraged to connect these contigs into scaffolds. Subsequently, assembled scaffolds exceeding 100 base pairs in length were aligned to EBV strain reference genomes, including HQ020558_GD2_NPC- tumor_China_2009 and KF373730_M81_NPC_China_1970 strains. GD2 was acquired as a minor subset of sequence data from next-generation sequencing of the complete DNA sequences originating from a biopsy specimen of NPC [27], and M81 was a valid representative of the EBV strains that were found in Chinese NPC [28].

### Statistical analysis

Association between EBV load and tumor size was examined by Spearman correlation test. Kruskal-Wallis H test was performed to compare baseline EBV load among cohorts with different responses. Logistic regression analysis was performed between plasma EBV DNA load and response, as well as being adjusted by other basic characteristics, including age, gender, stage, performance status, and previous treatment lines. We stratified quantitative variables such as TNB at different thresholds to identify their association with clinical outcomes. Kaplan-Meier analysis along with log-rank test were used to estimate the survival. Hazard ratios (HRs) were calculated using Cox proportional hazards regression model. R software (version 3.3.2) was used for statistical analysis. A two-tailed *p* value < 0.05 was considered statistically different.

## Results

### Patient cohort

Sixty-eight patients (45 in camrelizumab trial and 23 in nivolumab trial) were included in EBV-DNA analysis, and 60 patients with available tumor issues (42 in camrelizumab trial and 18 in nivolumab trial) were included in WES analysis. All baseline characteristics were displayed in Table 1.

**Table 1:** Patients’ demographics and baseline clinical characteristics

No. (%)	All patients (68)
Median age, years (range)	46 (23–69)
Sex	
Male	53 (77.9)
Female	15 (22.1)
ECOG performance status score	
0	23 (33.8)
1	45 (66.2)
Smoking	
Never	48 (70.6)
Former/current	20 (29.4)
Stage	
Primary metastasis	11 (16.2)
Recurrent with distant metastasis	57 (83.8)
WHO histological classification	
Undifferentiated non-keratinised	57 (83.8)
Differentiated non-keratinised	7 (10.3)
Keratinised squamous carcinoma	4 (5.9)
Distant metastasis sites	
Lung	40 (58.8)
Liver	41 (60.3)
Bone	31 (45.6)
Distant lymph	49 (72.1)
Others	10 (14.7)
None	1 (1.5)
Prior lines for advanced disease	
1	18 (26.5)
2	17 (25.0)
3	17 (25.0)
4 or more	16 (23.5)
Comorbidity	
Yes	41 (60.3)
No	27 (39.7)
Prior radiation	
Yes	57 (83.8)
No	11 (16.2)

ECOG: Eastern Cooperative Oncology Group; WHO: world health organization

### Association between EBV DNA titers and clinical outcomes

**Fig. 1:**
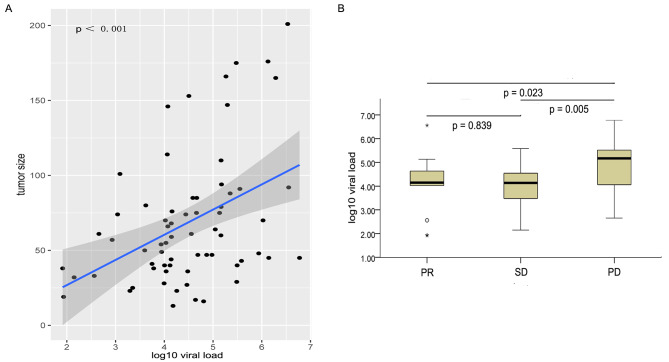
Baseline Epstein-Barr virus (EBV) load and clinical outcome. **A**. Spearman correlation between tumor size and plasma EBV copy number at baseline. **B**. Boxplot of log10 baseline viral load by Response Evaluation Criteria in Solid Tumors group. ORR, objective response rate; PD, progression disease; PR, partial remission; SD, stable disease

For 68 patients with baseline EBV DNA titers, the absolute viral load before immunotherapy was positively correlated with tumor size (spearman *p* < 0.001, Fig. 1A), and both PR and SD patients had significantly lower EBV load than PD patients (Fig. 1B).

**Fig. 2:**
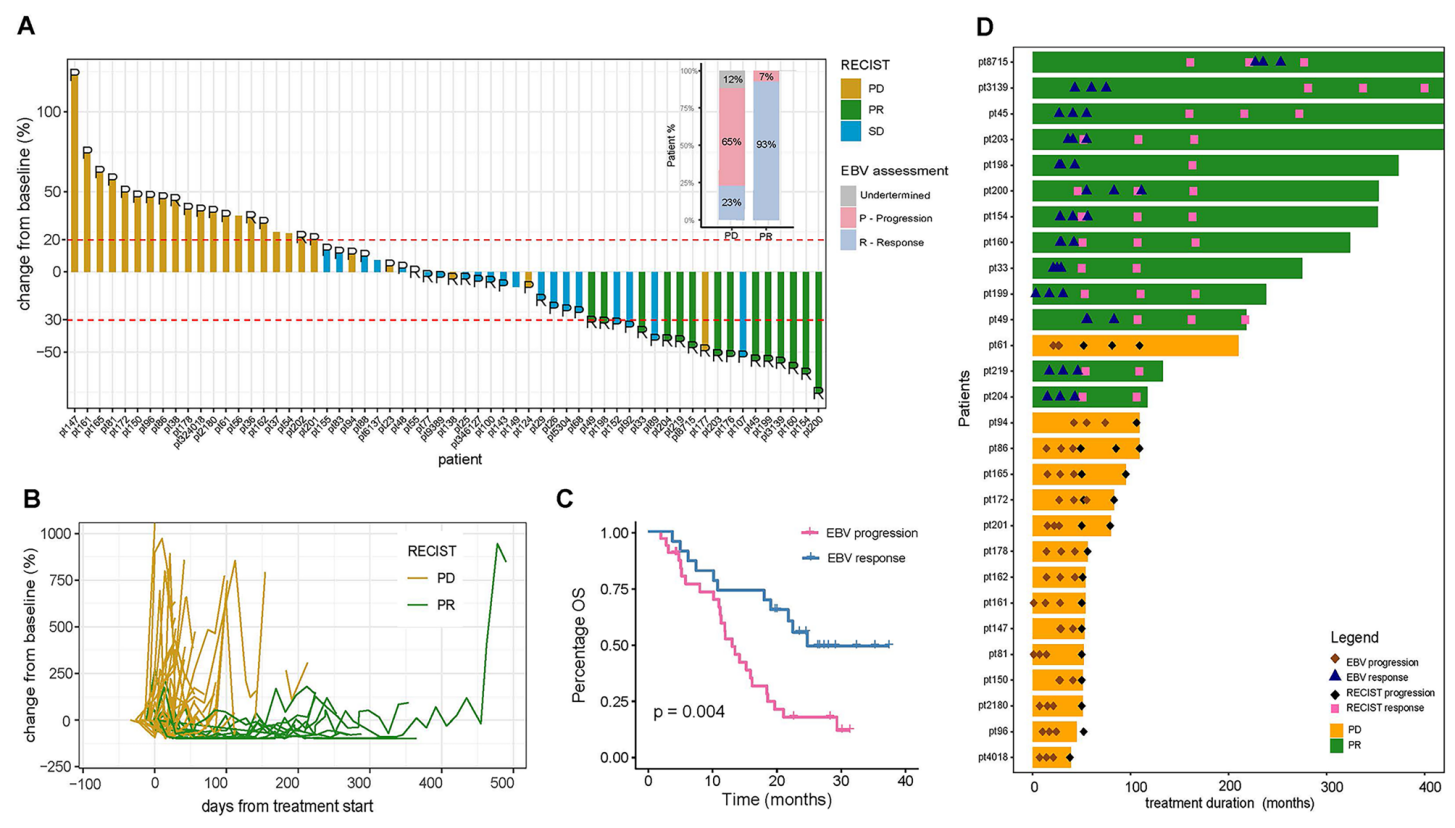
Change of EBV load during treatment and response to immunotherapy. **A**. Waterfall plot of change from baseline in tumor size for patients with nasopharyngeal carcinoma. **B**. Change from baseline in EBV viral load for patients with PD and PR. **C**. OS curve of patients with EBV response vs. those with EBV progression. **D**. Swimmer plot that shows time to initial EBV response/progression and RECIST response/progression in PR and PD patients. RECIST, Response Evaluation Criteria in Solid Tumors; PD, progression disease; PR, partial remission; SD, stable disease; EBV, Epstein-Barr virus; OS, overall survival

**Fig. 3:**
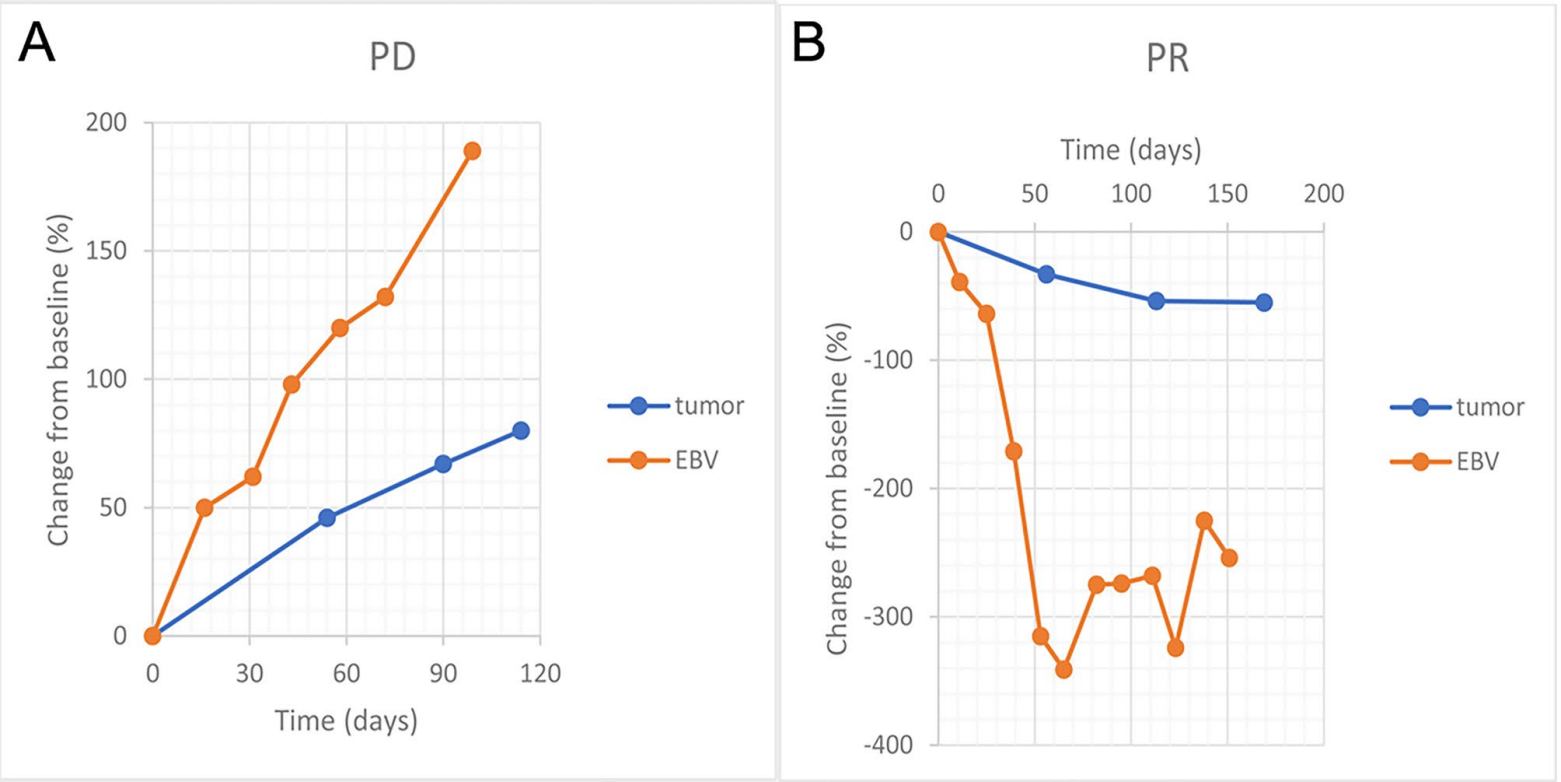
EBV and RECIST assessment of 1 PR and 1 PD patients. **A**. Tumor size and plasma EBV load change over time in a PD patient. EBV load was on log10 scale. **B**. Tumor size and plasma EBV load change over time in a PR patient. EBV load was on log10 scale. EBV: Epstein-Barr virus; RECIST: Response Evaluation Criteria in Solid Tumors; PD: progression disease; PR: partial remission

For the following analysis, we only included patients who had at least 3 post-baseline timepoints of EBV data and at least 1 post-baseline timepoint of radiographic assessment (*n* = 60). EBV assessment was highly consistent with RECIST evaluation (Fig. 2A). PR patients showed a stable or decreased viral load while PD patients displayed an increasing but highly fluctuating load within 50 days of treatment initiation (Fig. 2B). EBV assessment accurately distinguished patients who benefited from immunotherapy and those who did not. EBV responders (*N* = 23) had significantly improved OS (log-rank *p* = 0.004, HR = 0.351 [95% CI: 0.171–0.720], median 22.5 vs. 11.9 months) compared to the EBV progression patients (*N* = 33) (Fig. 2C, Figure S2A). A multi-variate analysis confirmed that the EBV assessment is independent compared to other clinical characteristics including sex, age, stage, ECOG performance status score and prior lines of treatment (log-rank *p* = 0.004, HR = 0.331 [95% CI: 0.158–0.696], Figure S2B). We compared the difference between the timing of EBV response/progression and RECIST response/progression in PR and PD patients (*n* = 28, Fig. 2D). The median time to initial EBV response was 28 days from the start of treatment whereas the median time to initial RECIST response was 53 days from the start of treatment. The median time to initial EBV progression and initial RECIST progression were 14 and 50 days, respectively. We included representative cases from 1 PD patient and 1 PR patient in Fig. 3 and the change curves revealed satisfactory consistency between radiographic evaluation and EBV load assessment.

### Association between molecular markers in WES data and clinical outcomes

The median TNB in 60 patients with WES data was 21. The distribution of TNB was not statistically different among patients with PR, SD or PD (supplementary Figure S3A) [see supplementary file]. Patients were divided into TNB high and low by median and no impact were showed on neither PFS nor OS between groups (supplementary Figure S3B, C) [see supplementary file].

**Fig. 4:**
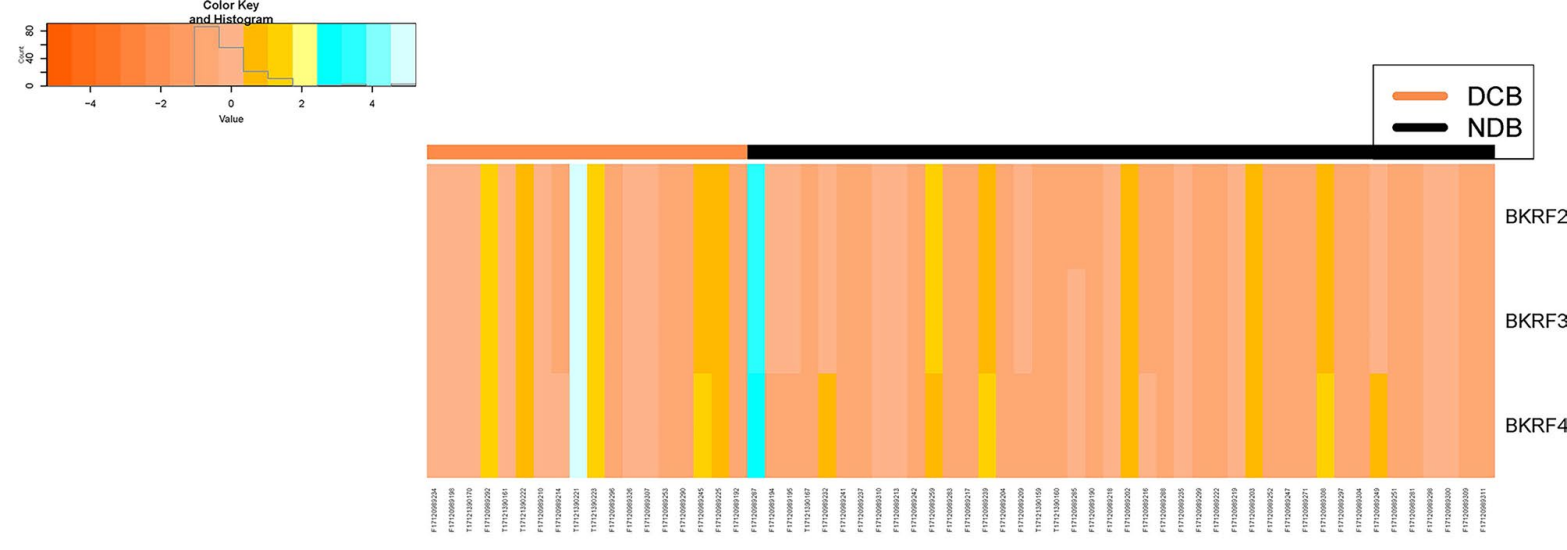
Levels of BKRF2, BKRF3 and BKRF4 in patients with durable clinical benefit (DCB) and non-durable clinical benefit (NDB)

**Fig. 5:**
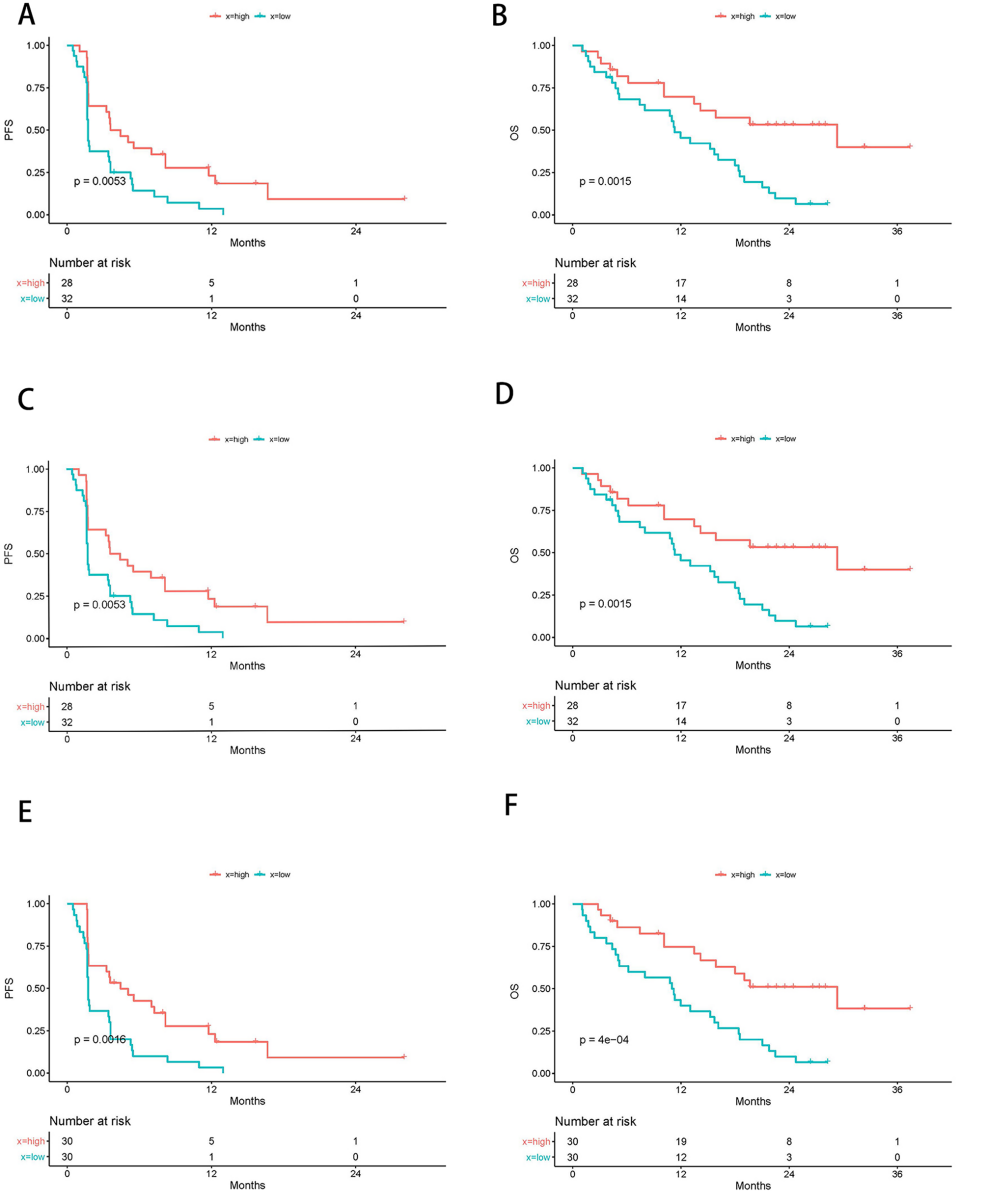
The effect of levels of BKRF2, BKRF3 and BKRF4 on patients’ progression-free survival (PFS) and overall survival (OS). PFS (**A**) and OS (**B**) curve stratified by median levels of BKRF2. PFS (**C**) and OS (**D**) curve stratified by median levels of BKRF3. PFS (**E**) and OS (**F**) curve stratified by median levels of BKRF4

Next, we explored the association between EBV lytic genomes, including BKRF2, BKRF3 and BKRF4, and clinical outcomes. Figure 4 showed that patients with DCB (*n* = 18) tended to have higher levels of BKRF2, BKRF3 and BKRF4 compared with patients with NDB (*n* = 42). The association between other genes and clinical efficacy were also analyzed as shown in supplemental Figure S4 [see supplementary file]. Levels of lytic EBV genes were grouped binarily as high versus low by median and further analyses demonstrated that high levels of BKRF2, BKRF3 and BKRF4 were associated with better PFS and OS (Fig. 5A-F).

In addition, we explored the association between EBV strains and clinical outcomes. EBV strains were determined by direct sequencing of EBV genomes in tumor tissues using WES. Our results showed that there were 23 and 25 EBV genome sequences mapped to known HQ020558_GD2_NPC-tumor_China_2009 [27] and KF373730_M81_NPC_China_1970 strains [28], and the others (*n* = 12) were not unique. We further compared the PFS and OS between GD2 and M81 EBV strains and the results showed no difference between groups (PFS: *p* = 0.13; OS: *p* = 0.66).

## Discussion

This study comprehensively investigated the role of plasma and tissue EBV biomarkers for predicting patients with RM-NPC receiving anti-PD-1 monotherapy. Overall, we found that patients with a lower baseline EBV DNA titer was positively correlated with smaller tumor size and better clinical response. Besides, EBV assessment was highly consistent with RECIST evaluation and patients with early clearance of plasma EBV DNA, namely EBV response, experienced superior survival outcomes. Furthermore, patients with high levels of BKRF2, BKRF3 and BKRF4 genes had significant longer median PFS and OS. These findings highlighted the value of plasma and tissue EBV biomarkers of anti-PD-1 therapy which could be incorporated into the guidance of individual treatment selection for patients with RM-NPC.

The role of plasma EBV DNA copy number as a biomarker for patients with RM-NPC receiving anti-PD-1 immunotherapy remains controversial. In POLARIS-02 study, the association of baseline EBV DNA titers with ORR was not statistically different but patients with RM-NPC with low baseline EBV copy number had a significantly higher DCB rate and improved survival [5, 20]. In our study, the baseline EBV load was positively correlated with tumor size and patients with PR and SD had significantly lower baseline EBV load than patients with PD, indicating that the blood EBV DNA might be derived from the tumor mass and could serve as an indicator of the tumor burden [29]. Previous studies have reported that large baseline tumor size was an independent prognostic marker of unfavorable efficacy in patients treated with pembrolizumab monotherapy [30, 31]. We speculated that the association between baseline EBV DNA level and clinical efficacy might result from the tumor burden. As for dynamic EBV titers, the Mayo Clinic Phase 2 Consortium study of nivolumab in RM-NPC observed a decreasing trend in EBV DNA titers in 87.5% of the responders, but found no association of plasma EBV DNA clearance with response rate and survival in RM-NPC patients receiving nivolumab [3]. However, positive results were observed in other reports [5, 19, 20]. A phase 3 trial (CAPTAIN-1st) showed that there was an association between the early clearance of EBV DNA and the response rate of camrelizumab in combination with gemcitabine and cisplatin as first-line treatment for RM-NPC [19]. In POLARIS-02 study, a large clinical study of toripalimab in RM-NPC patients, the ORR of patients with ≥ 50% decrease of plasma EBV DNA copy number on day 28 was significantly better than those with < 50% decrease. Additionally, improved survival was observed in patients with early decreases in EBV DNA titers [5, 20]. Here, we found that early change of EBV load, defined as EBV response/progression, was able to assess the treatment efficacy of anti-PD-1 monotherapy, with high consistency with RECIST evaluation. The change curve of EBV load assessment revealed satisfactory consistency with the curve of radiographic evaluation in PR and PD patients. Additionally, the median time to initial EBV response and progression were 25 days and 36 days prior to initial RECIST response and progression, respectively. The evaluation of response to anticancer therapy in patients with solid tumors presently depends on radiological assessments. Nevertheless, recurrent radiological evaluations are not devoid of constraints, such as increased radiation exposure for the patient and difficulties in assessing bone lesions [32]. Given the fact that the radiographic evaluation was difficult and inappropriate to be conducted so early and frequently in clinical practice, EBV assessment proved to be a convenient method for predicting the clinical efficacy of NPC patients receiving anti-PD-1 immunotherapy prior to radiologic review. Patients assessed as EBV progression who were unlikely to benefit from immunotherapy could be promptly scheduled for additional radiographic confirmation and expedited transition to new treatment. Moreover, patients who obtained EBV response had significantly improved survival than those who obtained EBV progression. These findings demonstrated that EBV assessment could predict clinical effects early and identify RM-NPC patients who could benefit from anti-PD-1 immunotherapy accurately. The monitoring of plasma EBV DNA levels during immunotherapy facilitated ongoing surveillance and enabled timely modification of treatment. To our knowledge, we are the first to use EBV assessment as a valuable tool for evaluating tumor response in patients with RM-NPC receiving anti-PD-1 monotherapy.

The lytic genes of EBV are less well characterized, unlike the latent genes of EBV, which have been studied extensively [33]. Here, we focused on the EBV lytic genes and investigated their association with clinical outcomes in RM-NPC patients treated with ICIs. Our results demonstrated that patients with high levels of BKRF2, BKRF3 and BKRF4 obtained long-lasting clinical benefit. Latent infection, rather than lytic infection, is the predominant program of EBV infection in NPC and lytic EBV genes are expressed only in small islets of NPC cells [34]. The proportion of lytic/latent EBV genes in patients expressing lytic genes might have changed. There could be an elevation in both the levels of lytic and latent EBV genes. Another potential scenario involves the transition from latent to lytic state in malignant cells [35]. Most of the EBV-infected tumor cells harbor latent virus, so the lytic reactivation was considered as part of an oncolytic treatment repertoire [36]. The latent state of EBV infection switches off the expression of most viral genes with strong immunogenicity, which makes EBV invisible to the cellular immunity, especially the CD8^+^ T cell response [35]. Here, we hypothesized that patients with high levels of BKRF2, BKRF3 and BKRF4 were supposed to experience lytic reactivation from latency and release abundant lytic antigens, which were recognized by immune effector peripheral T cells, particularly CD8^+^ T cells awakened by ICIs [37], thus achieving clinical response. The understanding of EBV-specific host immunity in RM-NPC patients receiving an-ti-PD-1 immunotherapy remains to be elucidated and our hypothesis also needs further validation.

There were some limitations in current study. First, the data were based on 2 phase I clinical studies with limited sample size and the risk of patient selection bias should be noticed. Second, the mechanisms of EBV DNA load and lytic EBV genes predicting the clinical outcome of anti-PD-1 immunotherapy in RM-NPC patients were unclear and needed further investigation.

## Conclusions

In summary, this study provided valuable EBV biomarkers predictive for RM-NPC patients’ response to anti-PD-1 monotherapy. Our results showed that the baseline EBV DNA load corresponded with clinical response and EBV assessment could predict clinical effects prior to radiographic evaluation and identify RM-NPC patients who could obtain long-term clinical benefit from anti-PD-1 monotherapy. We demonstrated that early clearance of plasma EBV DNA load and high levels of lytic EBV genes, including BKRF2, BKRF3 and BKRF4, were associated with improved survival. Future prospective studies with larger sample size are required to validate our findings.

## Electronic supplementary material

Below is the link to the electronic supplementary material.

Supplementary Material 1

Supplementary Material 2

Supplementary Material 3

Supplementary Material 4

Supplementary Material 5

## Data Availability

The data of this study are available upon reasonable request from the corresponding author, H Zhao.

## Abbreviations

RM-NPC: Recurrent or metastatic nasopharyngeal carcinoma
Anti-PD-1: Anti-programmed cell death 1
EBV: Epstein-Barr virus
PR: Partial response
SD: Stable disease
PD: Progression disease
OS: Overall survival
PFS: Progression-free survival
ICI: Immune-checkpoint inhibitor
ORR: Objective response rate
TNB: Tumor neoantigen burden
RECIST: Response Evaluation Criteria in Solid Tumors
DCB: Durable clinical benefit
CR: Complete response
NDB: Non-durable clinical benefit
PCR: Polymerase chain reaction
WES: Whole-exome Sequencing
HR: Hazard ratio
WHO: World health organization
