# Correction to: Early change of plasma Epstein-Barr virus DNA load and the viral lytic genome level could positively predict clinical outcome in recurrent or metastatic nasopharyngeal carcinoma receiving anti-programmed cell death 1 monotherapy

**DOI:** 10.1186/s12885-026-16657-0

**Published:** 2026-08-12

**Authors:** Shaoyan Lin, Huaqiang Zhou, Gang Chen, Jinhui Xue, Qianwen Liu, Jianing Li, Yanhua Yang, Yuanyuan Zhao, Hua Bao, Yan Huang, Yuxiang Ma, Hongyun Zhao

**Affiliations:** 1 Department of Clinical Research, State Key Laboratory of Oncology in South China, Guangdong Key Laboratory of Nasopharyngeal Carcinoma Diagnosis and Therapy, Guangdong Provincial Clinical Research Center for Cancer, Sun Yat-sen University Cancer Center, Guangzhou, 510060 P. R. China; 2 Department of Medical Oncology, State Key Laboratory of Oncology in South China, Guangdong Key Laboratory of Nasopharyngeal Carcinoma Diagnosis and Therapy, Guangdong Provincial Clinical Research Center for Cancer, Sun Yat-sen University Cancer Center, Guangzhou, 510060 P. R. China; 3 Geneseeq Research Institute, Nanjing Geneseeq Technology Inc, Nanjing, China

**Correction to: BMC Cancer 24, 797 (2024)**

**https://doi.org/10.1186/s12885-024-12564-4**

Following publication of the original article [1], the author reported a typographical error in the Results section, where the value “222.5” should read “22.5,” and that Figs. 2 and 3 must be swapped. The image currently presented as Fig. 2 corresponds to the legend and content intended for Fig. 3, and vice versa.

1. Typographical error in Results section.
  Original sentence:
    “EBV responders (*N* = 23) had significantly improved OS (log-rank p = 0.004, HR = 0.351 [95% CI: 0.171–0.720], median 222.5 vs. 11.9 months) compared to the EBV progression patients (N = 33) (Fig. 2C, Figure S2A).”
  Corrected sentence:
    “EBV responders (*N* = 23) had significantly improved OS (log-rank p = 0.004, HR = 0.351 [95% CI: 0.171–0.720], median 22.5 vs. 11.9 months) compared to the EBV progression patients (N = 33) (Fig. 2C, Figure S2A).”
2. Figures 2 and 3 published in the wrong positions (need to be exchanged)

Incorrect Fig. 2

**Fig. 2:**
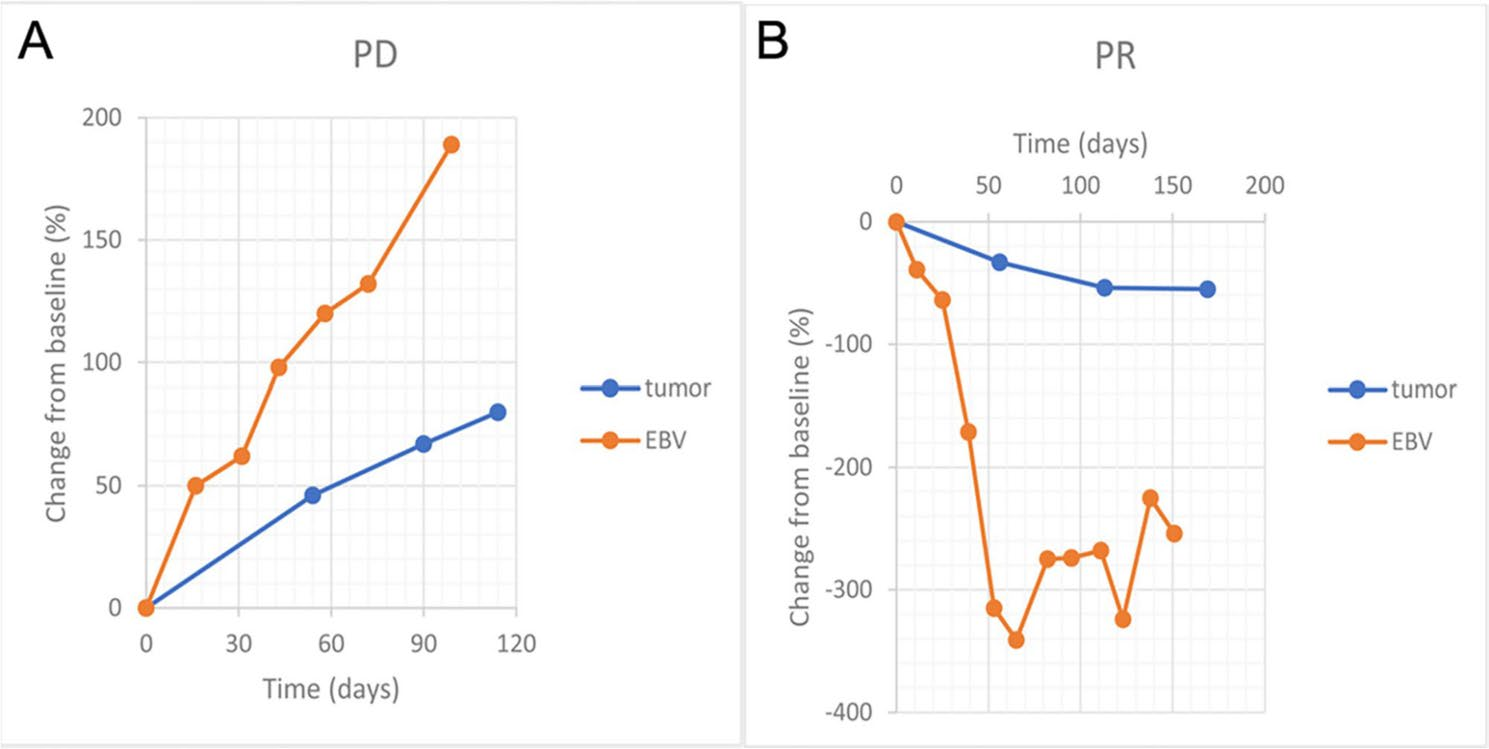
Change of EBV load during treatment and response to immunotherapy. **A**. Waterfall plot of change from baseline in tumor size for patients with nasopharyngeal carcinoma. **B**. Change from baseline in EBV viral load for patients with PD and PR. **C**. OS curve of patients with EBV response vs. those with EBV progression. **D**. Swimmer plot that shows time to initial EBV response/progression and RECIST response/progression in PR and PD patients. RECIST, Response Evaluation Criteria in Solid Tumors; PD, progression disease; PR, partial remission; SD, stable disease; EBV, Epstein-Barr virus; OS, overall survival

Correct Fig. 2

**Fig. 2:**
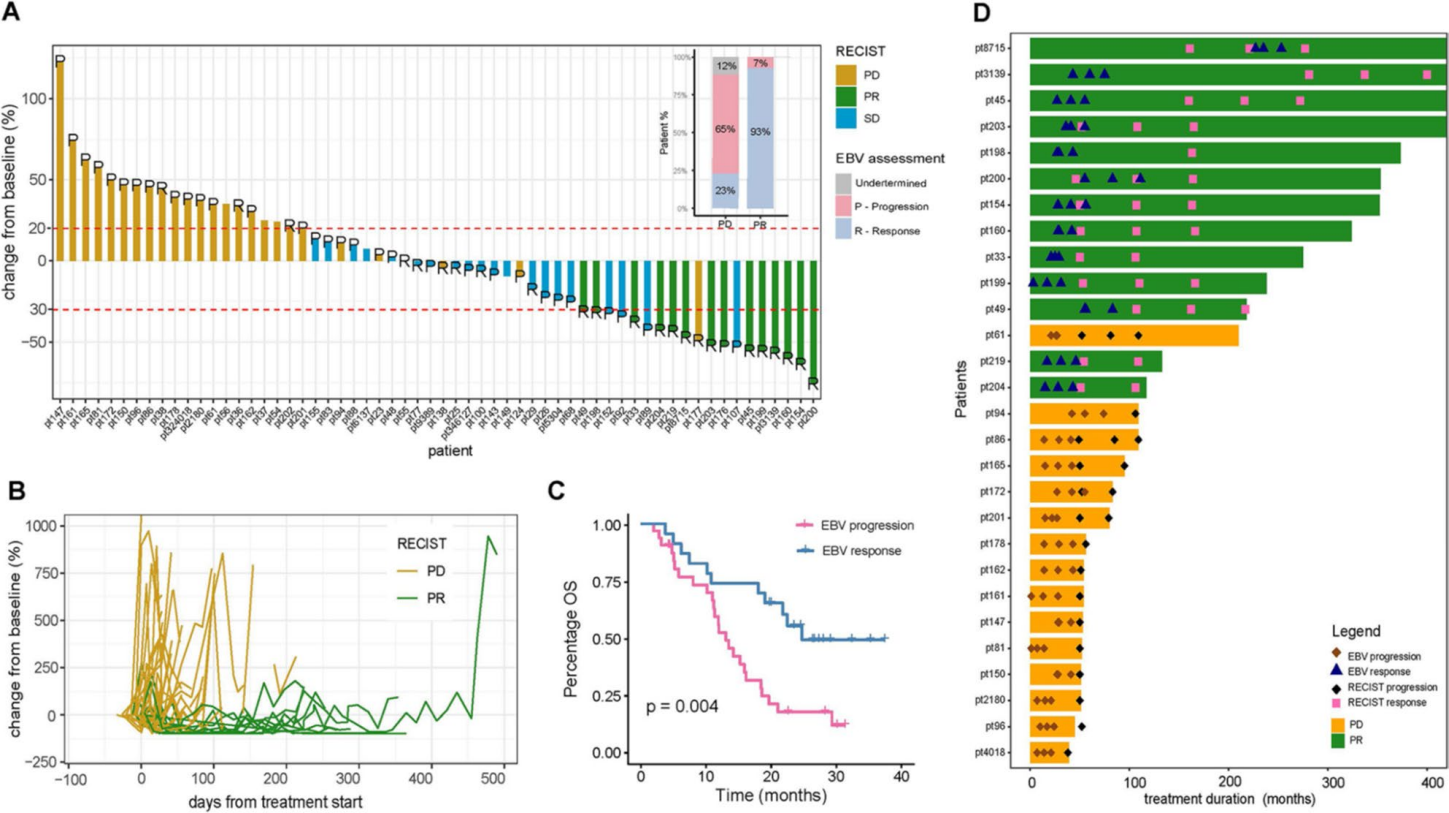
Change of EBV load during treatment and response to immunotherapy. **A**. Waterfall plot of change from baseline in tumor size for patients with nasopharyngeal carcinoma. **B**. Change from baseline in EBV viral load for patients with PD and PR. **C**. OS curve of patients with EBV response vs. those with EBV progression. **D**. Swimmer plot that shows time to initial EBV response/progression and RECIST response/progression in PR and PD patients. RECIST, Response Evaluation Criteria in Solid Tumors; PD, progression disease; PR, partial remission; SD, stable disease; EBV, Epstein-Barr virus; OS, overall survival

Incorrect Fig. 3

**Fig. 3:**
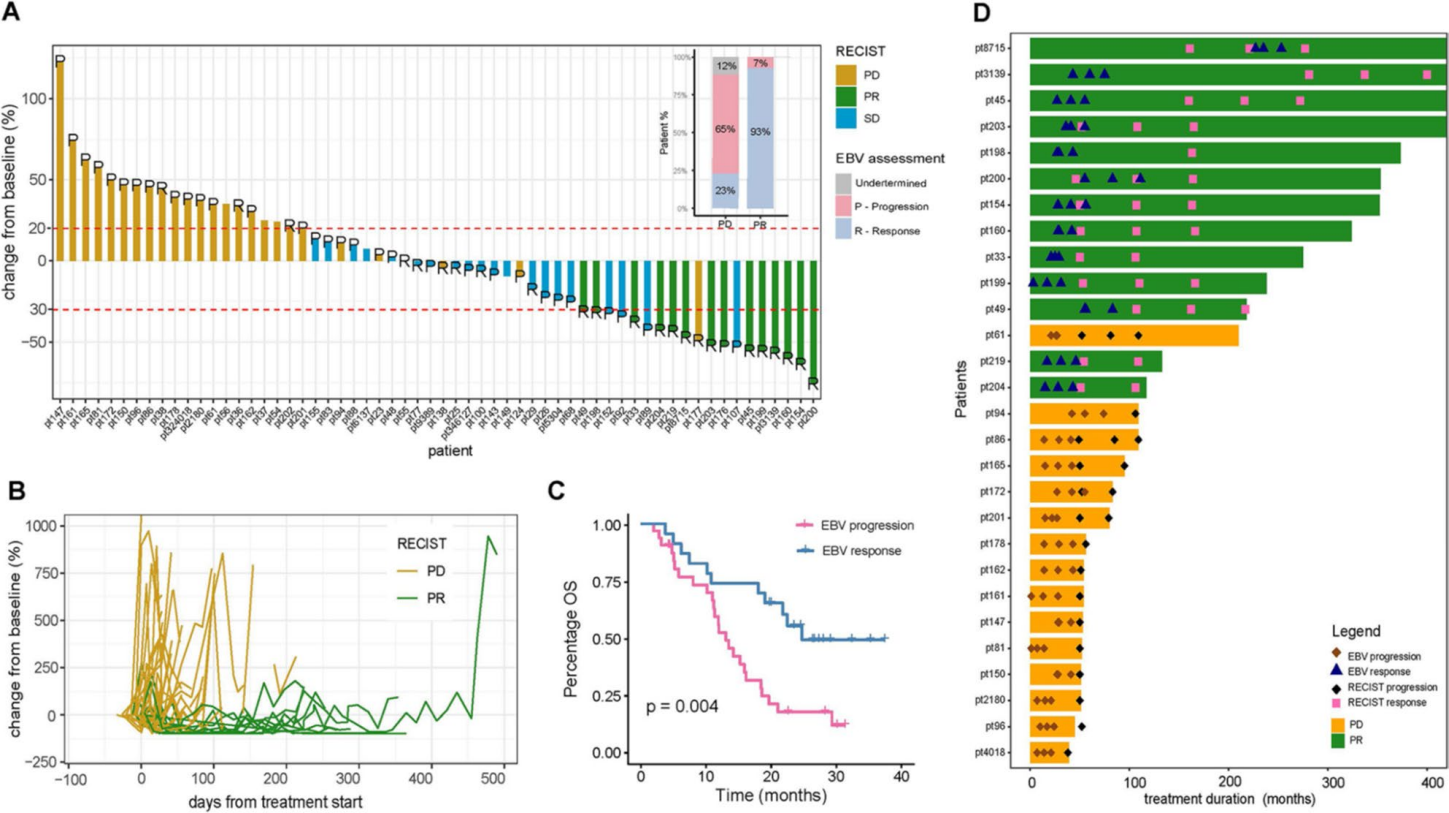
EBV and RECIST assessment of 1 PR and 1 PD patients. **A**. Tumor size and plasma EBV load change over time in a PD patient. EBV load was on log10 scale. **B**. Tumor size and plasma EBV load change over time in a PR patient. EBV load was on log10 scale. EBV: Epstein-Barr virus; RECIST: Response Evaluation Criteria in Solid Tumors; PD: progression disease; PR: partial remission

Correct Fig. 3

**Fig. 3:**
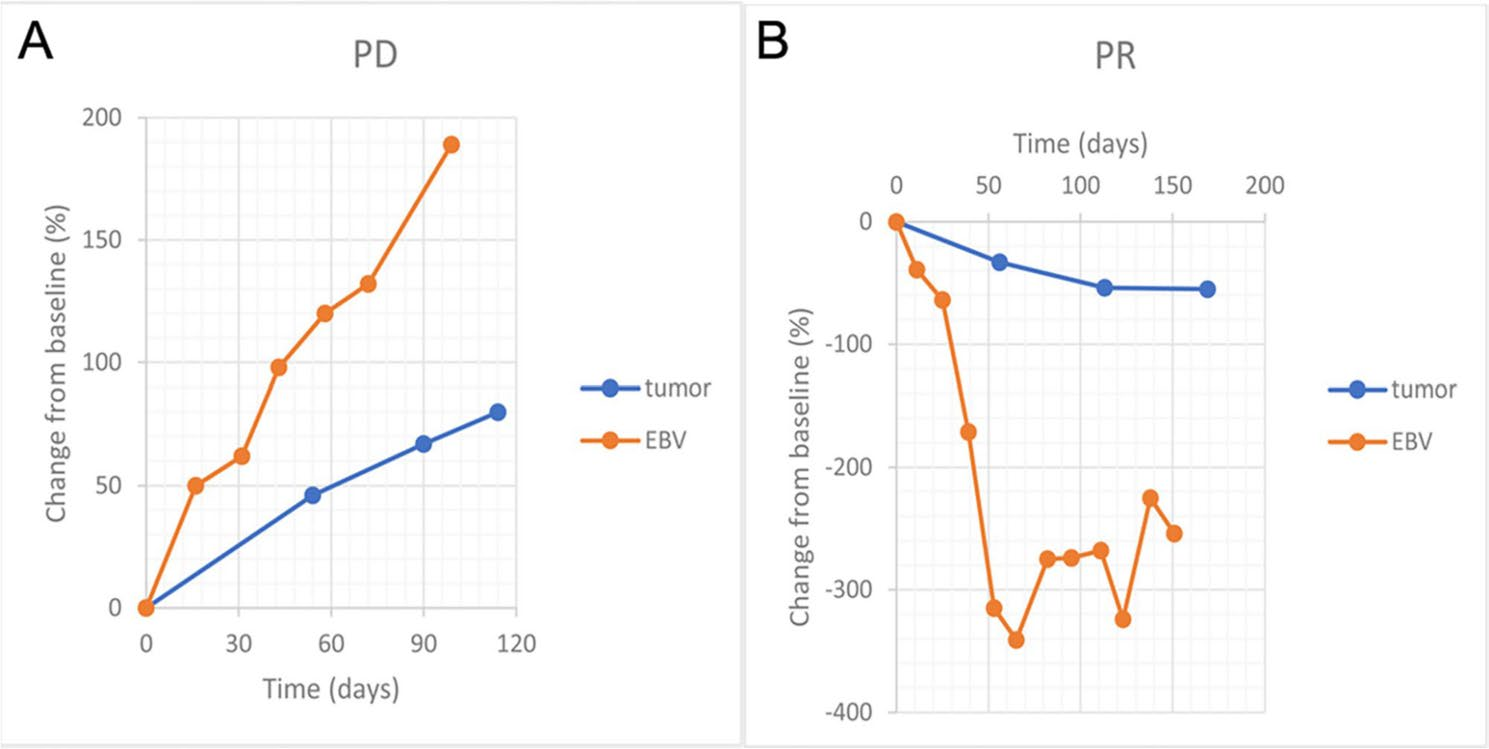
EBV and RECIST assessment of 1 PR and 1 PD patients. **A**. Tumor size and plasma EBV load change over time in a PD patient. EBV load was on log10 scale. **B**. Tumor size and plasma EBV load change over time in a PR patient. EBV load was on log10 scale. EBV: Epstein-Barr virus; RECIST: Response Evaluation Criteria in Solid Tumors; PD: progression disease; PR: partial remission
