# Supplementary figures and images for: Early change of plasma Epstein-Barr virus DNA load and the viral lytic genome level could positively predict clinical outcome in recurrent or metastatic nasopharyngeal carcinoma receiving anti-programmed cell death 1 monotherapy
Source: BMC Cancer. 2024 Jul 3;24:797. doi: 10.1186/s12885-024-12564-4 (PMC11223362; doi:10.1186/s12885-024-12564-4)

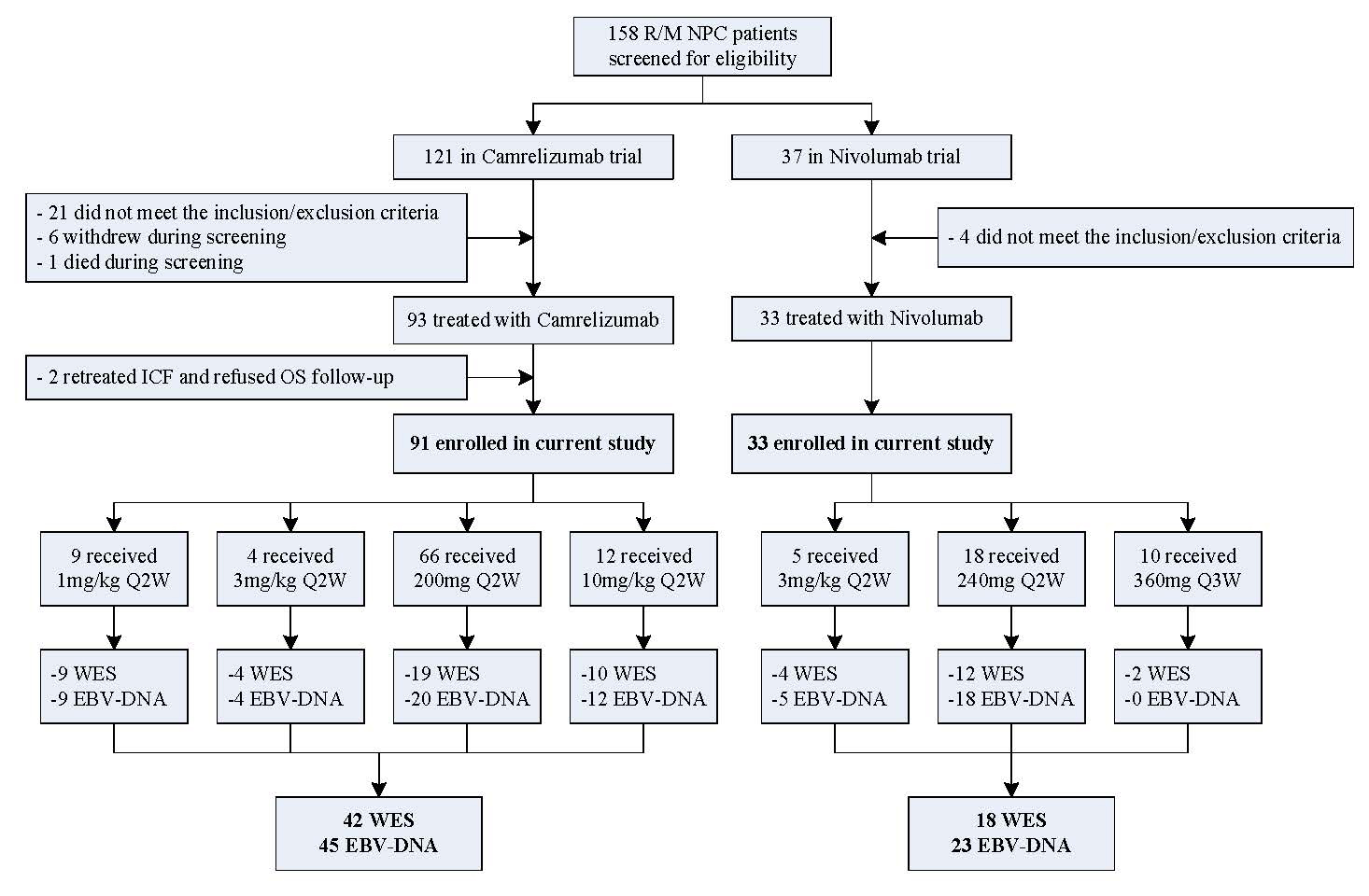

Supplement: Supplementary file 1 — Supplementary Material 1 [file 12885_2024_12564_MOESM1_ESM.jpg]

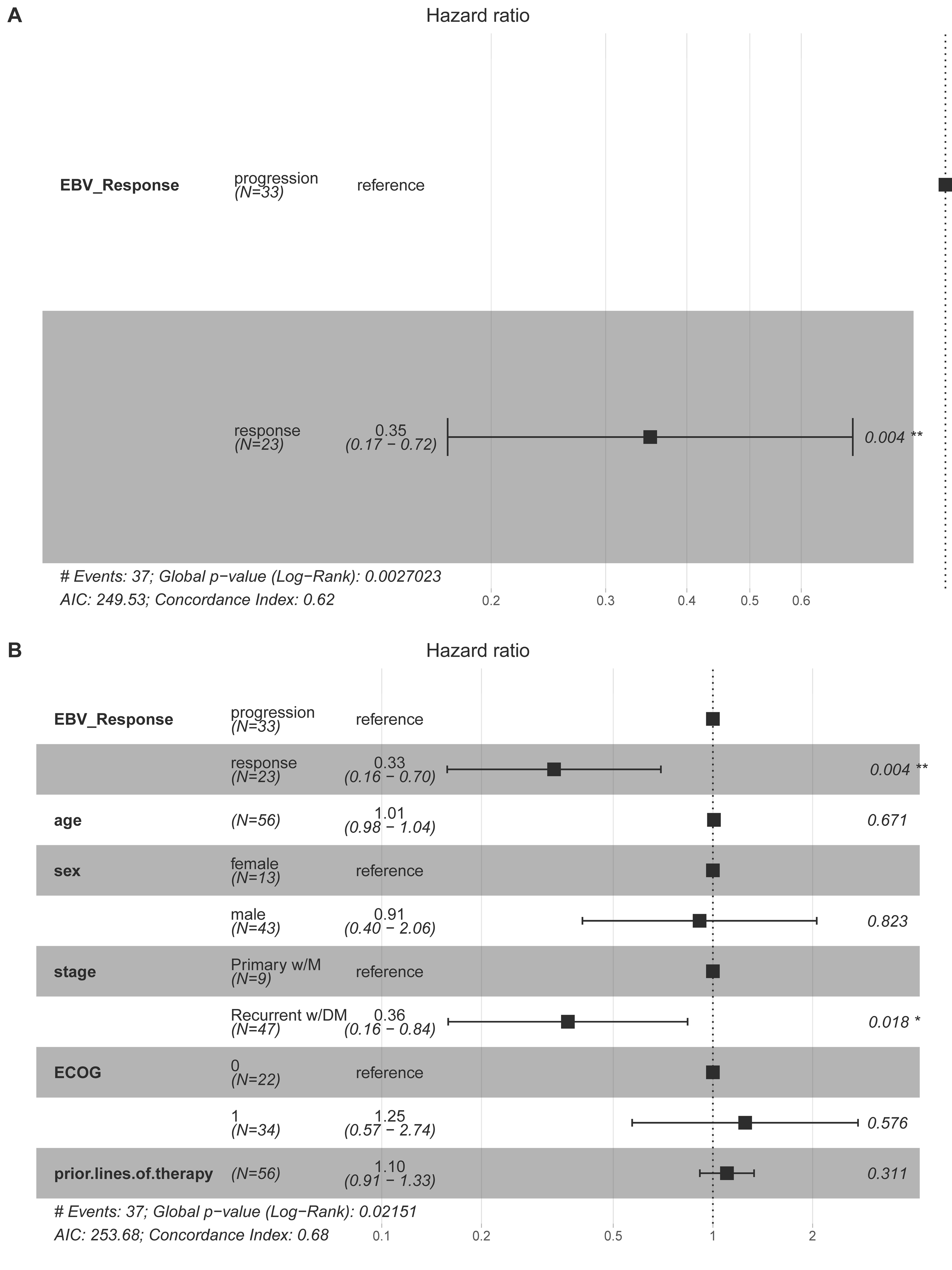

Supplement: Supplementary file 2 — Supplementary Material 2 [file 12885_2024_12564_MOESM2_ESM.jpg]

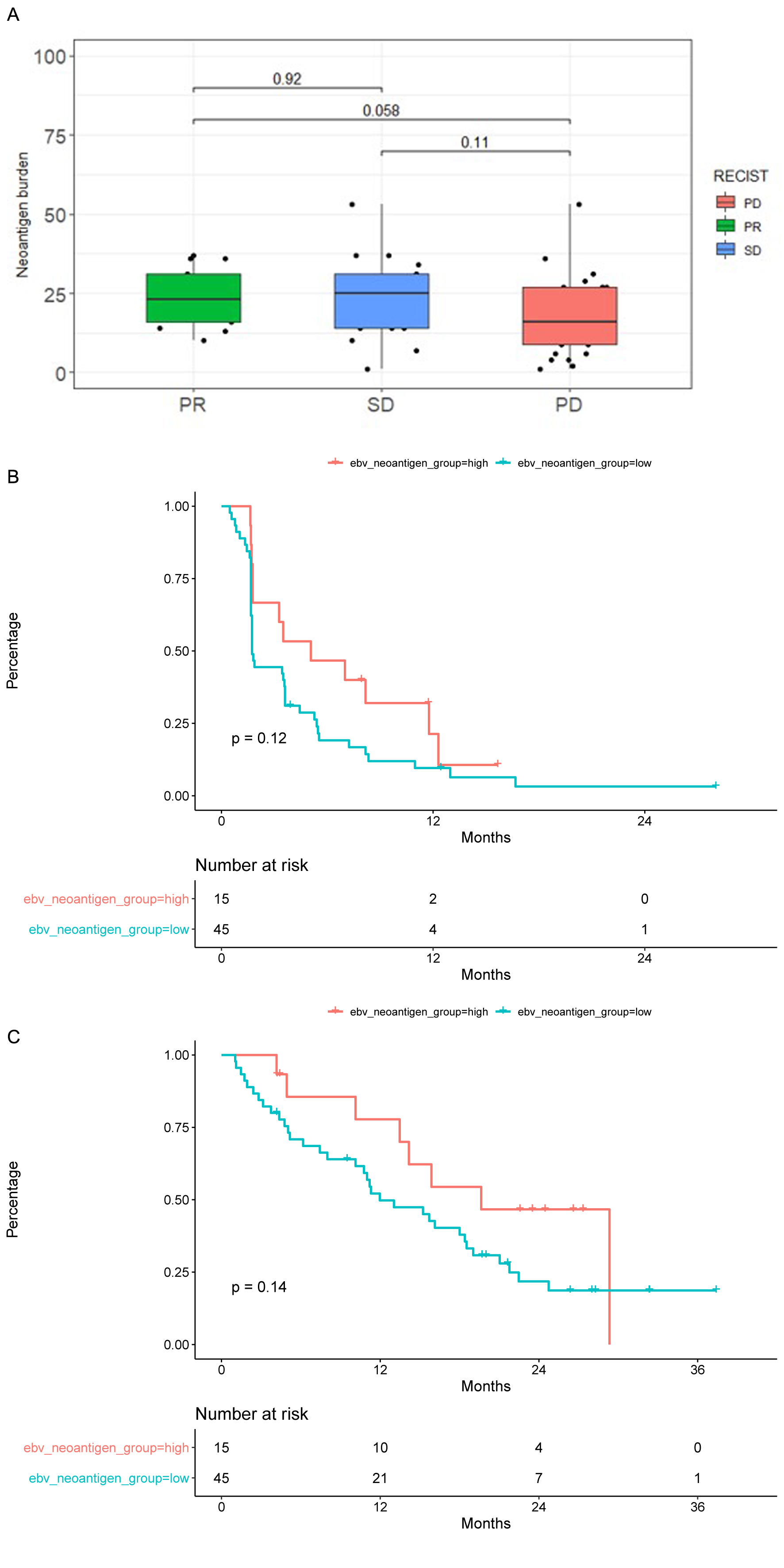

Supplement: Supplementary file 3 — Supplementary Material 3 [file 12885_2024_12564_MOESM3_ESM.jpg]

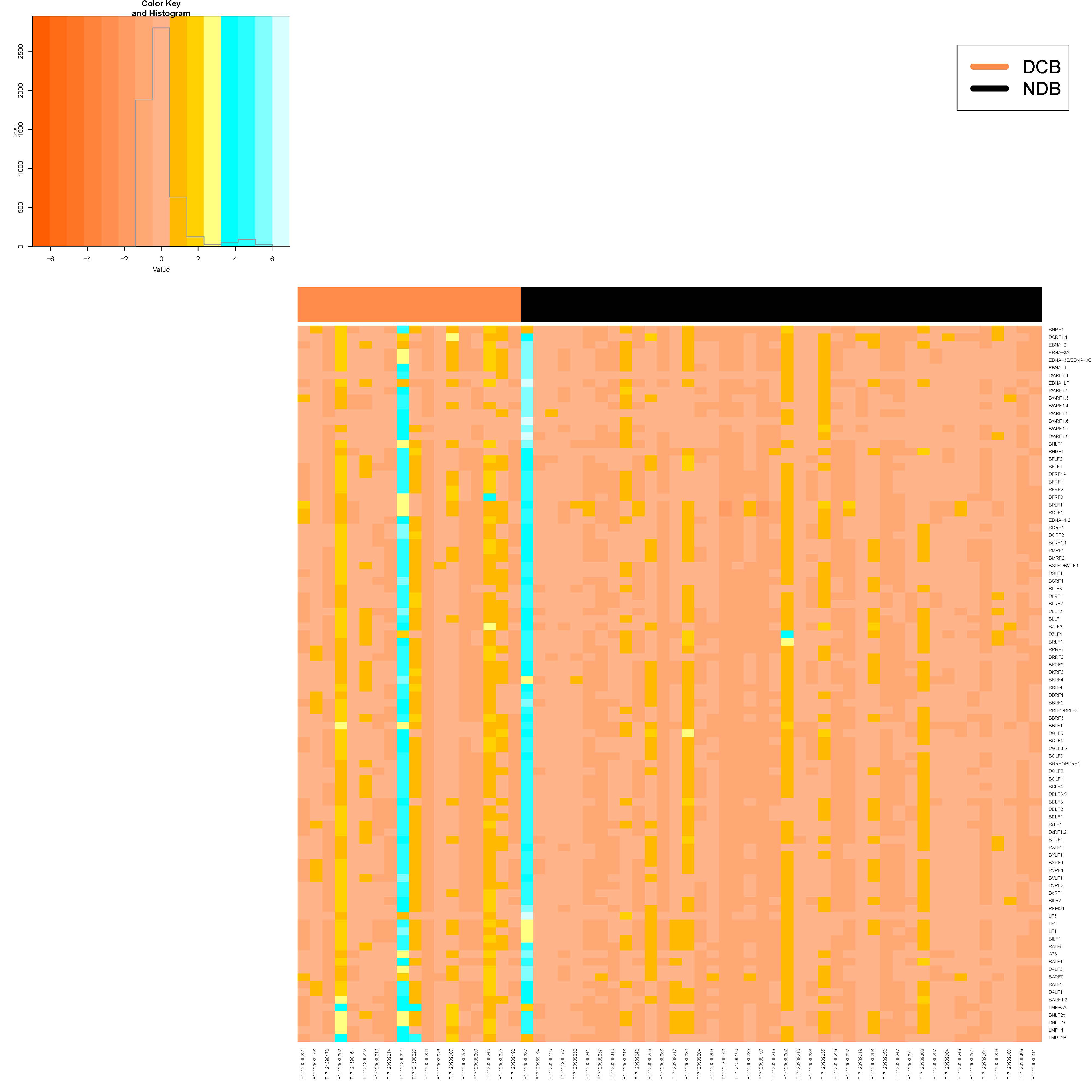

Supplement: Supplementary file 4 — Supplementary Material 4 [file 12885_2024_12564_MOESM4_ESM.jpg]
