# Supplementary material for: Early change of plasma Epstein-Barr virus DNA load and the viral lytic genome level could positively predict clinical outcome in recurrent or metastatic nasopharyngeal carcinoma receiving anti-programmed cell death 1 monotherapy
Source: BMC Cancer. 2024 Jul 3;24:797. doi: 10.1186/s12885-024-12564-4 (PMC11223362; doi:10.1186/s12885-024-12564-4)
Supplement: Supplementary file 5 — Supplementary Material 5 [file 12885_2024_12564_MOESM5_ESM.docx]

**Supplementary material**

**Figure S1.** The distribution of treatments and the screen process of participants.

**Figure S2.** The impact of EBV response on patients’ overall survival. Forest plots of **(A)** univariate analysis of EBV response and **(B)** multivariate analysis including EBV response, age, sex, stage, ECOG, and prior lines of therapy.

**Figure S3.** The effect of tumor neoantigen burden (TNB) on patients’ responses and survival. **A**. Distribution of TNB in 60 patients. PFS (**B**) and OS (**C**) curve stratified by median TNB. PD, progression disease; PR, partial remission; SD, stable disease; PFS, progression-free survival; OS, overall survival.

**Figure S4.** Levels of genes in patients with durable clinical benefit (DCB) and non-durable clinical benefit (NDB).
